# On the Methods for Obtaining a Natural History of Diseases

**Published:** 1846-04

**Authors:** Thomas Laycock


### III. ON THE METHODS FOR OBTAINING A NATURAL HISTORY OF DISEASES.

By Thomas Laycock, m.d.

IN A LETTER TO THE EDITOR.

York, Feb. 13th, 1846.

My dear Sir,—When I received the reprint you were so kind as to send to me of your admirable article entitled "Homœopathy, Allopathy," &c., I had already read it *in situ* with unmingled satisfaction. In compliance with the request you make on the fly-leaf for the opinions of your friends on the matters discussed in it, I send these to you with a very sincere and humble *quantum valeat,* and with a wish that they may aid in the reformation you seek.

I would here, as a general preliminary, and once for all, make a thousand apologies for any dogmatism or too confident expression of opinion that I may appear to be guilty of. Indeed I would rather make ten thousand here (if you think ten thousand necessary) than occupy your time and patience with a premonitory palaver and kotoo at every notion I may express. So you will really and truly understand that I dissent, assent, and assert with some diffidence and with humble deference to you especially, and to everybody else in general.

In the first place, then, I fully agree with you in all your propositions; I differ with you in two or three minor details only. With regard to proposition 4,* I advance as the result of my experience in vital statistics, that the numerical method is one not as yet *generally* applicable to medical observations; or, if generally applicable, only in points of detail so simple in character that they scarcely require it for their elucidation, except to those who wish for a numerical expression of facts. In the first place you want skilled observers; you want a regiment of *trained* men—trained, too, in the same school, at the same drill—so that, whether their observations be right or wrong, they will be alike right and wrong. You know well the difficulty of minute diagnosis; how much skill is required to tabulate effectually according to the inductive method (for this is the true principle of the numerical method) those cases of which you have no doubt; and under the most favorable circumstances for observation how many links in the chain of phenomena to be observed and classed cannot be made out at all. Pray observe I am not giving an opinion adverse to the numerical method, because I am sure it is a truly scientific method. What I advance is, that as yet it cannot be made *generally* applicable.

Another minor detail I object to is in proposition 19. You would abolish the practice of keeping and preparing medicines in the houses of practitioners. You never could do it. I have lived and practised in both rural and civic districts, and I give this opinion also as the result of experience. Thousands of practitioners will give you the same opinion. A much better arrangement would be to require every practitioner, whether physician or surgeon, living half a mile from a druggist's shop, to keep and prepare medicines in his house. Compel him to do it by law, that the health and comfort of his neighbours may be consulted—things they, at least, estimate more highly than his own sham notions of dignity. It is the selling of drugs and the drugging system that lowers the practitioner in public estimation. He might keep a warehouse full of drugs, and welcome, if he gave them all away, as *he ought to do.*

Nevertheless there is the germ of a right good reform in proposition

* "The general adoption by practitioners in recording their experience of the system known by the name of the *Numerical Method* is essential to the attainment of the ends proposed in the preceding paragraph, as well as in many that are to follow."

19. If generally adopted, it will give a heavy blow and great discouragement to the sectarian bigotry of medical men. The "pures" and the impures will be well mixed, and a glorious *tertium quid* will be the result of the operation. In Thomas Carlyle phrase, strong symmetrical forest trees will grow up, instead of clipped Dutch-garden dragons. Here an oak (a sound-headed, omniscient pure), there an ash, here an elm, there a wide-spreading beech (some Nestor of the general practitioners), or a chesnut; but all graceful, symmetrical things, each supporting each other when the storms come. Of course there will be stubble and brushwood—quacks and humbugs—but even from these may be extracted some good. If there be no good in them, then they are unavoidable evils under *all* circumstances, and must therefore be endured.

Proposition 17 is, I think, rather too strongly expressed.*

Having specified the objectionable points, I ought now in justice to specify the more valuable. This, however, is not an easy business. All the propositions contain pith and marrow. Perhaps the first is the most pithy; at least I shall stop at the first.† We are to "attempt to establish a true natural history of human diseases." As this is what I have been trying to do for some time, I will give you my views as to the method most likely to be successful. I premise, however, a special and very low "kotoo."

It is essential to the natural history required that there should be—1, a system of observation; 2, a system of classification. You cannot take up a jumble of observed things (such a jumble, for example, as the homœopathic cases), and by the mere force of labour in inductive analysis and synthesis deduce useful results. There must be a guiding thread of theory—a centre round which the facts may crystallize—an idea which shall determine the sides and angles. Well, then, as disease is an aberration from normal structure and function, a history of normal structure and function, or in one word the science of physiology must be the basis of your natural history of disease. You cannot adopt any other usefully or safely. Any other will lead you into the empiricism from which we want to escape.

The classification of diseases should, I think, be founded on *both* structure and function, but mainly on structure. Function is often dependent on structure. Structure, too, really varies less than function, and is therefore better adapted as a basis for that "more comprehensive and philosophical system of nosology" which you recommend in proposition 15. The mucous and serous tissues are those which present the strongest points of difference, and have been often adopted by taxonomists. My own method has been to take the embryological development of the tissues as the guide to observation and classification.

Twelve or fourteen years ago the blastoderma, or germinal membrane, was described as forming two layers of granules. From the outer of these (termed the serous layer) the nervous, osseous, muscular, and tegumentary systems of the body were said to be developed. The other or inner layer, situated next to the yolk, was termed the mucous layer. Between these, or from these, it was reported that the vascular layer was formed. From the combined changes which these two undergo, it was said the intestinal, respiratory, and glandular systems owe their origin. *All* these views may not be true, but the facts so described had the merit to me of being both useful and simple. I thought they formed a capital basis of classification. I was thus enabled, in treatment, to consider

* "No systematic or theoretical classification of diseases or of therapeutic agents ever yet promulgated is true, or anything like the truth, and that none can be adopted as a safe guide to practice." Some kind of guide is safer than no guide whatever; a classification of diseases is simply *a methodical arrangement of past experience:* this is always a *comparatively* safe guide.

† "To endeavour to ascertain, much more precisely than has been done hitherto, the natural course and event of diseases, when uninterrupted by artificial interference; in other words, to attempt to establish a true natural history of human diseases."

all the products of the serous layer—the muscles, bones, serous membranes, and skin (always excepting the glands embedded in it) as but one extended serous layer in different stages of development. Here is not only a leading idea for a comprehensive nosology, but also for a comprehensive pathology and therapeutics. You see nothing marvellous in the conversion of muscles and tendons into bone, or in the development of cartilage and bone in the plastic matter effused from serous membranes. The metastasis of disease in serous structures is just what you would expect; the sympathy and relation to remedies of organs distant as to site, but related as springing from the same tissue, is a matter of course. The pathology of the vascular system is perhaps better elucidated by this idea than that of the serous system. Embryologists report that it is compounded of both mucous and serous layers; but the serous evidently predominates in it. Pray note especially, first, its actual structure, and then those approximations to cartilaginous deposit in the subserous cellular tissue of the heart and arteries—the atheromatous deposit—so often observed. Pray note, also, the participation in metastasis from serous membranes; the decided sympathy of the heart and arteries with serous inflammation (the pulse is but little disturbed in true mucous inflammation); and the coincidence of a well-marked muscular and osseous development with the sanguine temperament, and a predisposition to rheumatic and arthritic affections. I assure you that I have found such an idea to be a valuable guide in practice, especially in those mixed affections of organs in which the mucous membrane is implicated, in consequence of disease of the subjacent serous layer; as in affections of the bronchi, for example.

I could multiply from the pathology of the skin and mucous membranes illustrations of the utility of taking a start in your natural history at the beginning. As, for example, the determining causes of the difference in the phenomena of epidemics. Why do people vomit in smallpox, but not in measles? Why are there pustules in the latter, and a rash in the former? What, in fact, determines the elective powers of the tissues for each specific virus? I really think that a grouping according to structure would solve many mysteries of this kind, as well as the mystery of secretion itself. Pray understand this is only a sketeh of the *kind of classification* I think best: every practitioner has his own method of grouping facts, and there might easily be a better than my method.

In attempting a natural history of diseases, the anatomy of structures and natural order or sequence of vital actions must require a large consideration. Nature cures, you say. How? What is her machinery? What her order and method? Does she restore to health in the same way as she begins and continues life? If so, we must inquire into her method, and the order of her acts, if we would lay the foundations of therapeutical science deeply. What is really the limit of the *vis medicatrix naturæ?* It may, perhaps, render the inquiry more simple to take a special example—suppose inflammation of a serous membrane. Some cause of disease has been determined to the pleura; its blood vessels are congested; they cannot be distended, as in the mucous or cellular tissues; and the first effort of the vis medicatrix of nature is to exude the watery portion of the blood, through the parietes of the vessels, constituting the primary grade or stage of inflammatory exudation. If this effort be successful the congestion is relieved, the effused fluid is absorbed, and there is an end of the matter. If it be unsuccessful, the effusion continues, but not to be absorbed. The fibrin now poured out contains cytoblasts, which are the germs of an organization analogous to that of the parent tissue. False membranes or connecting bands are now formed, and these become tendinous, cartilaginous, and even osseous. Nay, I have seen the whole of the costal pleura ossified. But are these products of disease to be considered marks of a vis medicatrix naturæ? I know you will readily allow that there are limits to the healing powers of nature. Science, assisted by close observation, will be able to fix those limits.

I think it may be assumed as a certain principle, that the *healing* effort differs in no degree from the *formative* effort. It is subject to the same laws, is effected by the same powers. The same may be said of the process of renewal, or moult, as Schultz terms it. These are as yet mysteries to us, although so much has been done by able microscopic observers. We are still uncertain as to the method by which regeneration or renewal of tissues takes place, and consequently we can scarcely attempt to determine the mode and limits by and within which the vis medicatrix operates. It is a great step gained to know how ignorant we are. The "cock-sure" practitioners, those heroes in physic, are the worst of all. And yet I never think of that vast amount of knowledge which the microscope and telescope have revealed to us without admiring delight. The sense of power which the intellect feels, as it sweeps from the infinitely grand in the universe to the infinitely minute in this microcosm of ours, is I think one of the highest and purest of pleasures. We are enabled to contemplate atomic beings here; there is the vast profound of mighty worlds. It is very clear, that in cell-life death is a necessary element; just the same thing occurs to the whole organism, in the structure of which these cellular beings play so important a part. The formative effort (the *nisus formativus* of the late venerable Blumenbach) contains within itself the elements of destruction. We have, therefore, to study a destroying as well as a healing power.

Function must not, however, be overlooked in the details of the natural system. In this field pathological chemistry will achieve triumphs as wondrous as those of microscopic research. The mucous layer of the embryo may here also be taken as the starting point, and its diverticula (the various glandular structures) be considered as parts of one great whole. The sympathies they manifest, the metastases they experience, the elective affinities they display, the vicarious action they occasionally assume, all present leading ideas for observation and classification. Groups of organs may also be established. I think of all organs the ovaria and testes exhibit the most extensive and general relations, and their influence in disease is perhaps the least understood, unless we except the muscular system. It is a remarkable circumstance that the physiology of the muscular system, and its pathological relations, should be so much neglected.

I have said that the *natural order* or *law of sequence* of vital phenomena is a point necessary to be known in constructing a natural history of diseases. We never can know the results of medication without a knowledge of the physiological antecedents; you are aware how much careful and earnest attention I have devoted to this point, and you know the result of my inquiries. It is simply this, that starting in the rudimentary germ, vital phenomena advance in waves of greater or less magnitude, the crest of the wave being the morbid period; that the periods of these oscillations vary in length from a few hours to several years; that they can be measured; and that they determine the periods, crises, and duration of diseases, and the order of their phenomena. As you have honoured me with a notice of my doctrines in No. XXXV. of your Review, I need only refer to it. In the history of the more common fevers, the utility of these doctrines is manifest enough. As they run through a known and determinate cycle, the action of remedies may be better estimated than in those in which the periods are not thrust before the eye of the practitioner. But even with regard to the more common forms, there are many "post hoc ergo propter hoc" absurdities committed. The natural and regular crisis or termination has often been triumphantly attributed either to some paltry medicine, a decillionth globule perhaps, or a few drops of hay tea; or to the effects of some heroic dose,—ten or twenty grains of calomel to wit—or a frightful drastic, in which case it was really nature winning a double triumph, conquering both the doctor and the disease.

It is very manifest that any medicine whatever (the more inert the better for the purpose) may be so administered that the number of recoveries, stated numerically, shall be more recommendatory of its activity than can be produced in favour of any other medicine. Suppose I was able in all cases of pneumonia to ascertain the day when the first rigor began, and suppose that I treated them all on the expectant method, but on the eve of the 7th or the 14th day I invariably administered one grain of rhubarb. The amendment on the next day would, in the majority of the cases thus treated, be so manifest that, without a knowledge of the law of sequence, the evidence in favour of the impotent grain, as an active and effectual remedy for pneumonia, would be absolutely irresistible. If amendment followed its administration in 28 cases out of 30, the probable ratio, (vide Fleischmann's table), how could you invalidate the "ergo propter hoc?" You could only answer, "your grain of rhubarb is like a literary pirate, it claims merit not its own. When the disease began then began also a series of changes, the purport of which was a restoration to healthy action."

Now on this point at least I belong to the *old* physic school, the real old school. The ancient physicians and their successors, studied the natural history of disease very closely, and applied this law very systematically to the treatment of febrile affections. Although I specify febrile diseases, please to remember that the course of *all* diseases is regulated by it, because all vital phenomena are. You may not always be able to trace it; several points are requisite for this, as undisturbed action in the organism, the power and habit of acute observation, manifest phenomena, and the like. But be certain that the natural history of all diseases will be fallacious unless this principle be made a leading idea in the observation and classification of pathological phenomena.*

I might refer for another illustration of the important bearing physiology has upon a true pathology to the phenomena of the nervous system. But we are really on the verge only of the vast field these present to us for observation and analysis, and I fear I should only weary you with long explanations; I therefore forbear. I think, however, I have said enough (I hope not too much) in support of my main argument. Yet would it be believed that there are able, nay, scientific, physicians who virtually, at least, prevent the application of physiology to the elucidation of pathology? You occasionally hear half-educated practitioners speak slightingly of him who devotes his leisure to physiological as well as to empirical reading. They hint that he is a "mere reader," "a theorist," "not a practical man," &c., but this is usually in the way of business; it is an ignorant dealer and chapman trying to extinguish a dangerous rival in the true artist. No great harm can result, as the pretender must needs yield at last. Not so, however, with the able physician; such an example as I met with not long ago in a weekly journal. The Editor, in reviewing a book on practical medicine, had animadverted in a passage (I think in the introduction) in which the author, an accomplished physician and a physiologist, observed that scientific men are not and cannot be

* Dr. Graves has published an interesting essay on the law of relapse periods in agues. The foundation of the essay is a case of quartan ague, which continued with intervals of health for more than two years. Dr. Graves found the relapses took place, with one or two trifling exceptions, exactly on the days on which a fit *would* have happened, provided they had recurred regularly. Now this law, which Dr. Graves publishes as a discovery, and of which this case of Dr. Graves' is a good demonstration, has long been known to me, and is simply a corollary deducible from my general law. It is, however, an interesting example of the utility of the latter, for by it Dr. Graves might have *predicted* the facts he observed so accurately.

practical, because they have had no experience. The Editor was "pulled up" by the offended author, and positively the latter, in his letter of reply to the criticism, repeated his offence.

It is obvious enough that a physiologist *and nothing else* cannot be a good physician; but a practitioner may be a physiologist and *something more.* While he studies the experiments of nature, he may have an acute vision for the minute shades of disease, and a quick perception of the effects of medicinal agents. Nothing will escape his prying eyes. He will be as fertile in curative resources as in theories; as keen a lover of practical information as of scientific knowledge. Further, this practical study of physiology is in itself most interesting. In effecting it you have not the trouble of torturing, and dissecting, and analysing. If, for example, you want illustrations of reflex action, your first patient will probably present them, look closely only. If not seen in your first, they will appear in your second; at all events, wait patiently, and you need not go and catch frogs or snails to vivisect. Indeed you may study the physiology of the nervous system everywhere, in everybody; in the man that walks before you in the streets, that stands before you in the pulpit, that struts his hour upon the stage, in people in the ball-room or at a dinner table; nay, you can make your bachelor friend, who pleasantly chats tête-à-tête with you at your fireside, wagging his leg the while, a subject of physiological research. All these, and a thousand more, are good examples of subjects for scientific investigation, presented by persons in perfect health. But when the proteiform diseases of poor human nature come before you, in all their varied phenomena, what an inexhaustible store of matter for observation and meditation !*

You will see that I think a physiological practitioner is the man of the age, and that it is of essential importance to the reformation you advocate that the practitioner should be a thoroughly good physiologist. He should have the science as ready at hand as his snuff-box. He should be *trained* to its use; for every symptom he should at least seek out, if he cannot find, a *rationale.* He should always be curiously catechizing physiology for the why and the wherefore. Much might be done in the training of the student by our clinical lecturers, if they would set their shoulders to the wheel; but, unfortunately, the empirical method is the easiest to teach as well as to practise. The student might be led on step by step. He might, for example, first have simple pathological problems in muscular action given to him. A man with morbus coxæ steps across the ward before a clinical class. Problem as follows:—Required to describe the bones and muscles entering into the deformity, and the rationale, physiological and pathological, of its production. Or if the students be more advanced, five patients are brought under the notice of the class; one is in an apoplectic fit, a second has scoliosis, a third has valvular disease of the heart, a fourth has phthisis, a fifth, pneumonia. Required to describe the peculiar characteristics of the respiration

* I cannot help giving you a small and homely illustration of these views, which has just occurred to me in looking over Dr. Ranking's excellent Half-yearly Abstract. At p. 72 of vol. ii, you will find two modes of treating scabies, the one the consequence of physiological and microscopical research, the other of empiricism. Now, firstly, scabies gets into very respectable families, and is no jest when a guest with them; 2dly, sulphur, its best cure, stinks. Mr. Stiff, a "*practical* man," and evidently of the right sort, namely, a physiological practitioner, meditates on these two points, and then on the disease. Scabies depends on an insect, the acarus: so the microscope tells us; the acarus belongs to a class of insects that breathe by tracheæ: this entomology sets forth. Comparative physiology shows that (like men), if insects have their tracheæ stopped up, they die. Now, Mr. Stiff argued that if the acari were smeared over with an oleaginous substance, their tracheæ would be stopped up, and they would be suffocated; consequently that, after all, it is the lard, and not the stinking sulphur, which cures the itch. He tried the simpler method, and has cured more than forty cases with simple inunction alone. The other example is the *empirical* use of veratria alba, solemnly published without a reason in the Annales de Thérapeutique. Now this, as well as half a dozen other *poisons of the acarus,* have been known empirically to vagrants from time immemorial.

in each case, and to give a physiological and pathological rationale of them. Would not this method sharpen the diagnostic and therapeutic wits, and be at least one safe and sure step towards a Natural History of Disease? Alas, for the "practical men," those "dealers and chapmen," that sneer in the way of their *métier* at physiologists as "mere theorists," and "bookworms!" What a rout a few swarms of students so trained would create among them!

Well, then, such being the leading ideas that should regulate reformation in medicine, you inquire, can they be brought into action? No! most emphatically; in the present condition of the profession it is impossible. You want a complete organization of the profession, so that there may be unity of action with a systematic division of labour. Medicine is eminently a science of assiduous incessant observation—not observation by one man, but by many, on a definite plan. But how can you get this requisite unity of action from the scattered units of the profession, when the public hospitals with their immense advantages have hardly yet approximated to a scientific league? Again, *non omnia possumus omnes.* Let me ask how many of the leading physicians and surgeons of the metropolis are skilled in microscopic philosophy or pathological chemistry? I know your answer. No practitioner engaged in only moderately extensive practice *can* be.

Well, then, what is to be done? Shall we give up the attempt in despair? Surely not. That course would be equally cowardly and impolitic. Impolitic, because the intelligent public is treading fast on our heels. People already begin to say, "when we send for a doctor he can only tell us what we know already;" or they slily hint to you how *they* would attain to successful practice—"I would always let nature cure the disease." It is certain that we cannot stand still; and if we determine to march forward, our only alternative is AN ENTIRELY NEW ORGANIZATION OF THE PROFESSION.

I see on reviewing my letter that I have made a considerable détour. From transcendental physiology to wrangling, jangling, medical reform is rather a long step downwards. But the regular current of my ideas cast up this cross-grained subject; it is quite a legitimate consequence of my views. You cannot separate medical organization from medical doctrine and discipline. The present disjointed condition of the profession may certainly be termed in some sort an organization; but it is such an organization as leads to disunion instead of union—to isolated efforts instead of combined action—to strife instead of peace. It is necessary that the twenty thousand general practitioners of the United Kingdom should enter cordially into the proposed reformation of medical science and practice; I say *necessary;* for without the aid of that intelligent and numerous body all efforts at amendment must be fruitless. But what cordiality can exist between the scientific leaders of the profession and the men in the ranks, when all of the latter, (being at least nine-tenths of their number) who practise midwifery are set down in the rules and regulations of their college as an inferior class of workmen? Can it be possible that these medical legislators have ever held a council to consider how they could best advance medical science and art? I think not, or surely this principle would not have been adopted. On the other hand, I think the English practitioners have pursued a mere shadow. The fellowship of the College of Surgeons can do them no good; its practical advantages are only nominal; its professional value is nil, unless it can be used to humbug the public. But does the title really signify that its possessor is a wiser or more skilful man than his neighbour, the "member"? Put *that* to the vote amongst the generals, and an immense forest of hands speaks out the negative. Do let us try and extinguish lies, and cant, and quackery within the profession, before we sally forth to rout the sham friends of suffering humanity without. But enough on these hateful squabbles. Neither the present status nor the proposed reforms are worth contending for; they both must lead alike to nothing.

My own notion is, that the inductive method should be applied to our internal economy; and why should not a scientific body bring scientific principles of government into daily action? The profession living in each of the large towns, and in the surrounding districts, should be incorporated, and have all the privileges and responsibilities of our civic corporate bodies. The influence of numerous medical corporations dotted over the country, if their action were confined to their own locality only, would be considerable; but if they acted conjointly through a general council, what potent results might we not expect! You need only refer to the occupation abstract of the population returns to see that there would be no deficiency as regards numbers. Five years ago, the physicians and surgeons in five provincial towns stood thus:—Birmingham 166, Bristol 192, Leeds 130, Liverpool 334, Manchester (including Salford) 274. Now you cannot give me any solid reason why these large bodies of medical practitioners should not be incorporated. You cannot say that the discipline, dignity, or progress of the profession, would be retarded by such incorporation. You cannot say that they have not a right to the aid of the law in establishing a system of self-government, having in view the maintenance of professional honour, the advancement of medical science, and the public weal. Whatever argument you bring against my proposition applies equally to the medical corporations of the metropolis.

Pray note the advantages which would accrue from this scheme. Firstly, you would secure unity of action by a general council, constituted by representatives from the several corporations. You could secure a joint purse and division of labour in the sciential evolution of the profession. Every medical corporation would have its library, its reading-room, its museum for pathology and the kindred sciences, its curator, its physiological investigator, as well skilled in microscopic as in biochemical analysis. And who knows but that rich and enlightened laymen, seeing our efforts, would join us? Indeed, they certainly would, and the several local corporations would be enriched by legacies for sciential purposes. Public-spirited men would think more of the prevention of disease, by increase of knowledge, than of the cure of disease by increase of hospitals. With such a system I should indulge a hope that before long every practitioner would keep a case-book on a uniform plan, and each corporation would have its statist to work out the results by the numerical method.

I might as well stop here, but permit me to add that I augur good from the movement you have commenced. I trust confidently that it will usher in a true reformation of the profession. I hope we shall all soon be convinced that our political advancement, as an industrial body, depends upon our sciential advancement as a profession; upon the good we can effect; upon the knowledge we can gain; upon, in short, our moral and intellectual condition. I hope, too, that the miserable rivalry of grades will cease, or at least so much of it as will allow of unity of action upon these principles. This united action of the profession is indeed a worthy object of ambition; the more difficult of attainment, the more glorious when accomplished. It would surely be a noble sight to see our army of twenty thousand practitioners (now a disorganized multitude) marching forward in disciplined battalions, obedient to a gradation of officers, turning aside from no obstacles, winning triumph after triumph, as well over ignorance and error as over social prejudice and ingratitude, and setting an abiding example to mankind of the power of science to gain permanent worldly power and effect real objects. Is there any reason why we should not try? Of course the word impossible must be excluded from our vocabulary.

Believe me, my dear Sir, yours most faithfully,

T. Laycock.

